# Dermoscopic findings in Tinea Capitis among under 18 children in dermatology polyclinic patients: a hospital-based cross-sectional study

**DOI:** 10.1097/MS9.0000000000001530

**Published:** 2023-11-17

**Authors:** Ahmed Isse Ali, Abdisalam Ibrahim Aden, Abdirahman Khalif Mohamud

**Affiliations:** a Department of Dermatology, Mogadishu-Somali Turkey Training and Research Hospital; b Faculty of Medicine and Health Sciences SIMAD University, Mogadishu, Somalia

**Keywords:** Dermoscopic finding, East Africa, Mogadishu, Somalia, Tinea Capitis, *Trichophyton sudanense*, *Trichophyton violaceum*, under 18 children

## Abstract

**Background:**

Tinea capitis is a fungal infection that affects the scalp. It is caused by a group of fungi known as dermatophytes, which thrive in warm and moist environments. In Somalia, there is a data shortage regarding dermatological conditions, especially in Mogadishu, the most populous city in the country. Tinea capitis has gone unreported despite its high prevalence in Somali dermatology clinics and the Somali diaspora in Western countries. The absence of up-to-date information hampers the capability to diagnose, treat, and prevent Tinea capitis. Therefore, the study aims to evaluate dermoscopic signs about isolated organisms and potassium hydroxide (KOH) examination.

**Method:**

A hospital-based cross-sectional study was implemented between January and April 2023 in Mogadishu, Somalia. All eligible Tinea capitis-infected children were included in the study. Microscopically, analysis was conducted by adding 10% of KOH in fungal elements. Data were analyzed using descriptive statistics and the χ^2^ test at *P* value less than 0.05.

**Results:**

A total of 76 tinea capitis-infected children participated in the study; 56% were age group between 5-9 years old, 68.4% were male, and 92.1% showed KOH positivity. *Trichophyton violaceum* (65.8%) and *Trichophyton sudanense* (14.5%) were the most common fungal organisms detected in the culture. comma hairs (93.10%), scales (40.80%), and corkscrews (32.90%) were the most common dermoscopic signs of tinea capitis. The demographical characteristics and dermoscopic signs of tinea capitis significantly associated with the positivity of KOH examination were age, sex, comma hairs, corkscrew hairs, broken hair, Scales, and Zigzag hair.

**Conclusion:**

Children in Mogadishu, Somalia, bear a significant burden of Tinea Capitis infections. *Trichophyton violaceum* and *Trichophyton sudanense* were the predominant causative agents identified in the cultures. The most common dermoscopic signs of tinea capitis observed in this study were comma hairs, scales, and corkscrew patterns. Hence, early diagnosis of Tinea Capitis infections and timely, effective treatments with contact tracing are highly needed.

## Background

Highlights

- A total of 76 tinea capitis-infected children participated in the study; 56% were age group between 5-9 years old, 68.4% were male, and 92.1% showed potassium hydroxide positivity.
- Trichophyton violaceum (65.8%) and Trichophyton sudanense (14.5%) were the most common fungal organisms detected in the culture. Comma hairs (93.10%), scales (40.80%), and corkscrews (32.90%) were the most common dermoscopic signs of tinea capitis.
- The demographical characteristics and dermoscopic signs of tinea capitis significantly associated with the positivity of potassium hydroxide examination were age, sex, comma hairs, corkscrew hairs, broken hair, Scales, and Zigzag hair.

Tinea capitis, also known as ringworm of the scalp, is a fungal infection that affects the scalp. It is caused by a group of fungi known as dermatophytes, which thrive in warm and moist environments^1^. This condition is most found in children and does exhibit a strong gender bias, although it can affect individuals of all ages^2^. Clinical presentations of tinea capitis can range from mild symptoms such as scalp flaking and discoloration to more severe manifestations, which include an itchy rash, hair loss, and the development of small, black dots on the scalp^3^. This condition is contagious and can spread from person to person through direct contact with an infected individual or via contact with contaminated objects like combs, brushes, hats, or pillows^4^. Fortunately, tinea capitis can be effectively treated with either oral or topical antifungal medications^5^.

Fungi from the *Trichophyton* and *Microsporum genera* can both lead to tinea capitis. However, the primary causal agent varies across different geographic regions and experiences temporal fluctuations^6,7^. Various laboratory methods can be employed to confirm the diagnosis. Notably, some dermatophytes like *M. audouinii* and *M. canis* can be distinguished through their unique fluorescence under Wood’s ultraviolet light. Regrettably, *Trichophyton tonsurans* does not exhibit this fluorescence, rendering this tool ineffective^8^. In most cases, the responsible organism can be identified through a fungal culture on Sabouraud dextrose agar or Mycocel® agar, following a potassium hydroxide preparation of hair from the affected area. Samples for culture can be obtained by scraping with a scalpel or more conveniently by employing a cytobrush or a moistened cotton swab^9^. Dermoscopy is a noninvasive, swift, and cost-effective procedure with a well-established history of effectiveness as an ancillary technique for evaluating hair and scalp conditions. It has been recognized as a supportive technique for diagnosing tinea capitis^5^.

In Somalia, there is a data shortage regarding dermatological conditions, particularly fungal infection especially in Mogadishu, the most populous city in the country. Tinea capitis has gone largely unreported, despite its high prevalence in Somali dermatology clinics and the Somali diaspora in Western countries^4,10–12^. It is crucial to gain a comprehensive understanding of the current state of knowledge, specific disease patterns, dermoscopic indicators, and common causative factors in Somalia. It might enhance the treatment and preventive strategies. The absence of up-to-date information severely hampers the capability to diagnose, treat, and prevent Tinea capitis in Somalia. Therefore, the study aims to evaluate dermoscopic signs in relation to isolated organisms and potassium hydroxide (KOH) examination.

## Materials and method

### Study design and setting

A hospital-based cross-sectional study was implemented between January and April 2023. All Tinea capitis infection patients who had mycological testing, dermoscopic and clinical examinations of scalp lesions with the edge of a blunt scalpel, hair stumps were scraped off for direct microscopic examination and culture were included in the study after excluding individuals who are older than 18 years and those whose legal guardians refused to sign the consent form and participate in the study.

### Laboratory procedure

To identify the fungal elements, samples were placed on a glass slide, added with 10% KOH, and then microscopically analyzed. To examine the affected areas of the scalp, we used a portable dermoscope (DermLite II hybrid, San Juan Capistrano, California) and captured images directly through the dermoscope using an iPhone 12, following the acquisition of informed consent from caregivers or legal guardians of the children. The reliability of the photos in Figure 2 taken with the iPhone 12 is attributed to their consistent camera settings and precise geotagging information present in the metadata. The cultural experiment was conducted and incubated at a temperature of 25°C on Sabouraud dextrose agar supplemented with antibiotics, including 0.5 mg/ml of cycloheximide. These cultures were maintained for 4 weeks and were subject to periodic examinations to monitor potential microbial proliferation. Cultures were considered negative if no discernible growth had occurred. Identification of the microorganism was achieved through the utilization of Lactophenol cotton blue stain, employing an analysis of colony morphology as well as microscopic observations of the culture mounts^13^.

### Sampling technique and data collection procedure

This study included all Tinea Capitis infections in children under 18 years of age who were either admitted to or referred to the study hospital. Those who declined to participate were excluded. Instead of conducting a sample size calculation, we employed a post hoc power analysis as recommended by Kim *et al.* ^14^ This power analysis aimed for a statistical power of 80%, with an alpha level set at 0.05^15^. The research included 76 patients initially suspected of having tinea capitis, but following the administration of a KOH test, it was found that 7.9% tested negative for the condition. To collect the data Dermatology residents who had received training in dermoscopic or the recognition of dermoscopic indicators of Tinea capitis carried out the dermoscopic assessment. The images were examined, and the results were reported by a dermatologist with experience in hair diseases and training in the recognition of Tinea capitis-specific signs (hence referred to as the expert).

### Data analysis

The collected data were cleaned, coded, and entered on the speeded sheet imported into the SPSS version 20 (SPSS) for analysis. Data were analyzed by descriptive statistics and presented frequency with percentage for categorical variables and mean with maximum, minimum, and stander deviation (SD) for continuous variables. The χ^2^ test was used for full cells and Fisher exact test was used for cells expected less than 5 frequencies in 20% of the total cell at *P* value less than 0.05. Data were presented in tables and figures, and this work report aligns with the STROCSS criteria^16^.

## Results

### Baseline characteristics

A total of 76 tinea capitis-infected children participated in the study, 56% were age group between 5-9 years old, 68.4% were male, and 92.1% shows KOH positivity. In addition, 90.8% of the study participants showed fungal culture positive and *Trichophyton violaceum* (65.8%) was the most common fungal organism detected in the culture, followed by 14.5% *Trichophyton sudanense*, 7.9% *Trichophyton tonsurans*, and 2.6% *Microsporum audouinii* (Table 1).

**Table 1:** Baseline characteristics (*n*=76)

Characteristics	*n* (%)
Age	
5–9	43 (56.6)
10–16	33 (43.4)
Mean age=9.80, SD=3.21	
Sex	
Male	52 (68.4)
Female	24 (31.6)
KOH	
Negative	6 (7.9)
Positive (*n*=70)	70 (92.1)
Endothrix	61 (87.14)
Ectothrix	9 (12.8)
Culture	
Negative	2 (2.6)
Contaminated	5 (6.6)
Positive	69 (90.8)
Organism (*n*=69)	
*Trichophyton violaceum*	50 (65.8)
*Trichophyton sudanense*	11 (14.5)
*Trichophyton tonsurans*	6 (7.9)
*Microsporum audouinii*	2 (2.6)

KOH, potassium hydroxide.

### Dermoscopic signs of tinea capitis

**Figure 1:**
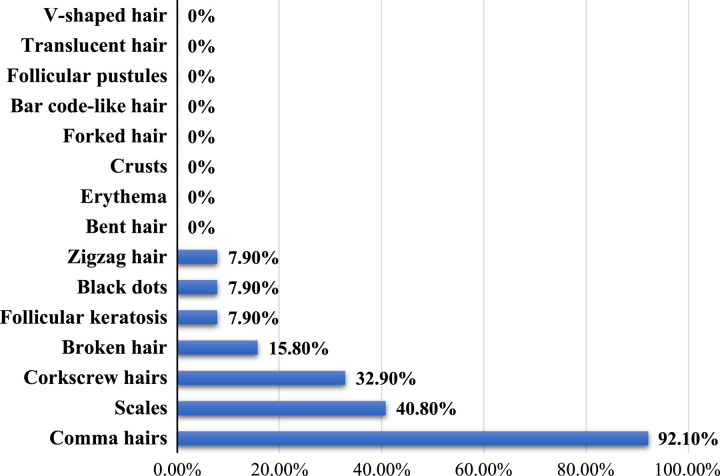
Dermoscopic signs of tinea capitis (*n*=76).

**Figure 2:**
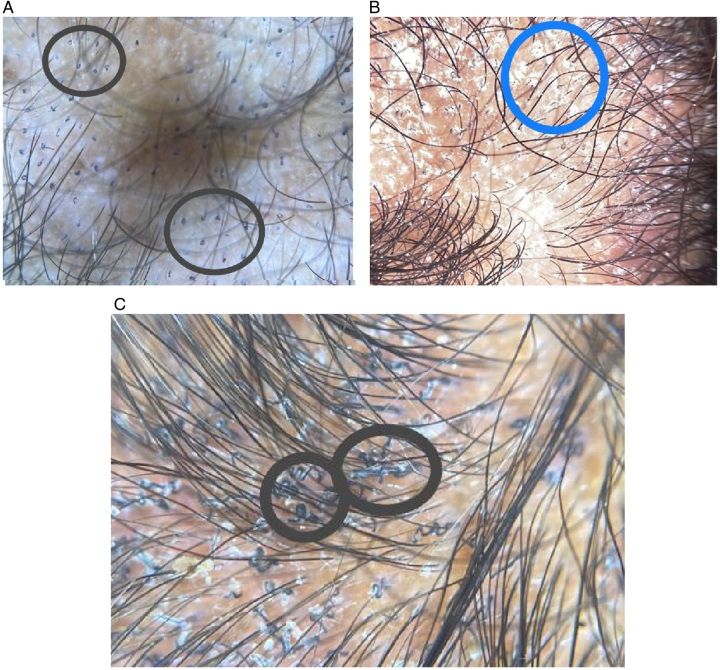
(A) Shows comma hair in the black circles, (B) picture that shows scales, (C) shows multiple corkscrew signs in the side and outside of the circles.

This study showed that the most common dermoscopic signs of tinea capitis were comma hairs (93.10%) followed by 40.80% of scales, 32.90% corkscrew, 15.80% broken hair, while Follicular keratosis, Black dots, and Zigzag hair showed 7.9% each of them and all other sings not detected (Figs. 1 and 2).

### Relationship between the KOH examination and demographical characteristics and Dermoscopic signs of tinea capitis

This study showed that the Age (*P* value=0.033) and Sex (*P* value=0.001) of the child have a significant association with the positivity of KOH examination. Moreover, comma hairs (*P* value <0.001), Corkscrew hairs (*P* value=0.001), Broken hair (*P* value <0.001), Scales (*P* value <0.034) and Zigzag hair (*P* value <0.001) were dermoscopic signs of tinea capitis that significantly associated with the positivity of KOH examination while Follicular keratosis and Black dots not significantly associated (Table 2).

**Table 2:** Comparison of KOH examination and demographic characteristics and Dermoscopic signs of tinea capitis (n=76)

	KOH examination			
Dermoscopic signs	Positive, *n* (%)	Negative, *n* (%)	χ2	*P*
Age				
5–9	37 (86.0)	6 (14.0)	4.999	0.033*,^a^
10–16	33 (100.0)	0		
Sex				
Male	52 (100.0)	0	14.114	0.001*,^a^
Female	18 (75.0)	6 (25.0)		
Comma hairs				
Present	70 (100.0)	0	76.000	<0.001*,^a^
Absent	0	6 (100.0)		
Corkscrew hairs				
Present	19 (76.0)	6 (24.0)	13.289	0.001*,^a^
Absent	51 (100.0)	0		
Broken hair				
Present	6 (50.0)	6 (50.0)	34.743	<0.001*,^a^
Absent	64 (100.0)	0		
Follicular keratosis				
Present	6 (100.0)	0	0.558	0.455^b^
Absent	64 (91.4)	6 (8.6)		
Scales				
Present	31 (100.0)	0	4.488	0.034*,^b^
Absent	39 (86.7)	6 (13.3)		
Black dots				
Present	6 (100)	0	0.558	0.455^b^
Absent	64 (91.4)	6 (8.6)		
Zigzag hair				
Present	0	6 (100.0)	76.000	<0.001*,^a^
Absent	70 (100.0)	0		

KOH, potassium hydroxide.

* Significant level at α=0.05.

a Fisher’s exact test.

b χ2 test.

## Discussion

This study enroled 76 individuals diagnosed with tinea capitis, and a remarkable 92.1% of them tested positive for fungal elements during the KOH examination, while only 7.9% yielded negative results. KOH preparations are a widely used method for diagnosing fungal infections, including tinea capitis. A KOH preparation is deemed positive for tinea capitis when microscopic examination reveals the presence of hyphae. When a sample of infected scalp hair is subjected to KOH treatment, it dissolves the keratin, a protein found in hair and nails, while leaving the fungal cells intact. These fungal cells can then be observed under a microscope, appearing as branching, filamentous structures known as hyphae. This finding conclusively confirms the presence of a dermatophyte infection, which is crucial for effective treatment. Similar studies have consistently reported an 88% KOH positivity rate^13^.

Furthermore, previous research has documented KOH false negative rates ranging from 5 to 40%. This can be attributed to the skill level of the diagnosing medical professional and limited medical equipment, especially in sub-Saharan African countries^17–19^. Therefore, it is strongly recommended that the diagnosis of tinea capitis patients rely not only on KOH testing but also on the clinical observation of experienced professionals.

The study revealed that 90.8% of participants tested positive for fungal culture, with *Trichophyton violaceum* being the most frequently isolated fungal organism. It was followed by *Trichophyton sudanense, Trichophyton tonsurans, and Microsporum audouinii*. Numerous studies have also reported *Trichophyton violaceum* as the most common cause of tinea infections^19–21^. In addition, various other fungal organisms, such as *Trichophyton verrucosum, Trichophyton tonsurans, and Trichophyton sudanense*, have been identified in different cohorts. American studies have highlighted *Trichophyton tonsurans* as the most prevalent causative agent, while European studies have reported a high incidence of both *Trichophyton tonsurans and Trichophyton violaceum* ^2,22^.

These variations can be attributed to geographical differences, where a specific fungal species may become more prevalent in certain areas. This phenomenon suggests the possibility of a transmission circle, as these fungi are highly contagious and can be easily spread from person to person through direct contact or contact with contaminated objects, particularly in warm and humid regions of the body where they thrive. Furthermore, poor hygiene practices, sharing personal items, and residing in crowded or humid environments are common contributing factors in the study area and across the entire African continent, exacerbating the situation.

The dermoscopic examination offers a simple and noninvasive procedure that serves as a valuable diagnostic tool for tinea capitis. This study investigates various dermoscopic indicators, with the most prevalent being “comma hairs,” accounting for an overwhelming 93.10% of the study participants. Comma hairs are distinguishable as twisted, coiled strands resembling a comma or question mark. They result from fungal infection disrupting the natural hair growth pattern and are frequently encountered during the initial stages of tinea capitis, although they might be absent in certain cases^23^. Furthermore, other research studies have identified scaling, either diffuse or perifollicular, as another common sign, which aligns with the second most prevalent dermoscopic sign recognized in this study^24^. In addition, our study reveals that broken hair and corkscrew signs rank as the third and fourth most common dermoscopic indicators, respectively. Several studies have reported that broken hair exhibits no significant correlation with the causative agent or type of invasion^2,22,25,26^. It is essential to note that scaling and broken hairs are not pathognomonic signs of tinea, as they may appear in various scalp fungi or other dermatological conditions^27^.

In this study area, there is a noticeable prevalence of reinfections of dermatophytosis, particularly among young individuals, which is frequently observed in dermatology clinics. We hypothesize that this trend is the result of a confluence of numerous risk factors including incomplete treatment, exposure to contaminated surfaces or objects, the sharing of personal items, suboptimal hygiene practices, and underlying health conditions that can heighten the risk of recurrence, particularly in individuals with compromised immune systems. It is worth noting that dermatophytes are not typically categorized as opportunistic fungal infections directly associated with immune compromise, as with Cryptococcus neoformans and Pneumocystis jirovecii pneumonia. Nevertheless, there remains a possibility that individuals living with compromised immune systems and chronic conditions might become more susceptible to various infections, including fungal ones^28–33^.

This study identifies that the age and sex of the child are significant demographic factors associated with a positive result in KOH examinations for fungal elements. Although prior studies were not directly compared, it is important to note that the prevalence of these infections can fluctuate based on age and various other factors. In general, tinea infections are more frequently observed in children than in adults, although they can affect both age groups. Furthermore, a positive KOH test does not necessarily imply an active infection, as fungal elements can persist in the skin or hair even after treatment. Therefore, in this study area, the diagnosis is not solely reliant on KOH positivity; it integrates clinical features into the interpretation of KOH test results and facilitates a more precise differential diagnosis. Notably, dermoscopic signs, such as comma hairs, corkscrew hairs, broken hair, scales, and zigzag hair were found to be significantly associated with KOH examination positivity in this study, whereas Follicular keratosis and Black dots showed no such association.

## Conclusion

Children residing in Mogadishu, Somalia, bear a significant burden of Tinea Capitis infections, with a notable prevalence among males aged 5–9 years. *Trichophyton violaceum* and *Trichophyton sudanense* emerged as the predominant causative agents identified in the cultures. Moreover, distinct features such as comma hairs, scales, and corkscrew patterns were commonly observed in the dermoscopic examination of Tinea Capitis cases within this studied population. Hence, there is a compelling need for the early diagnosis of Tinea Capitis infections, along with the timely implementation of effective treatments and a broader epidemiological investigation incorporating contact tracing. These measures are vital for curtailing disease transmission and enhancing both prevention strategies and overall quality of life. Furthermore, it is highly advisable to delve into the contributing factors behind the elevated prevalence of Tinea Capitis and to work towards enhancing healthcare accessibility for this demographic.

### Strength and limitation

It is the first similar study in Somalia focusing on Tinea capitis cases, which is a common paediatric dermatophyte infection, but it has been largely unreported in the region. The study is a cross-sectional design, which can establish associations between variables but cannot determine cause-and-effect relationships and inability to assess treatment outcomes. Despite this limitation, the study provides valuable information that can enhance treatment, interventions, and preventive programs for Tinea capitis in Somalia and the region as well.

## Ethical approval and consideration

The study aligns with the rules and regulations of the World Medical Association Declaration of Helsinki. Ethical approval was obtained from the Human Research Review Board of the Somali Türkiye Training and Research Hospital with approval IRB number: (MSTH/14849). All eligible respondents’ legal guardians explained the study objective and requested participation. A written informed consent was obtained from the patient’s parents/legal guardian for publication and any accompanying images. A copy of the written consent is available for review by the Editor-in-Chief of this journal on request. In addition, all legal guardians were informed that they had a full right to discontinue the study without penalty. Confidentiality was kept, the questionnaire was anonymous, and data was presented without reflecting individual information.

## Consent

All eligible respondents’ legal guardians explained the study objective and requested participation. A written informed consent was obtained from the patient's parents/legal guardian for publication and any accompanying images. A copy of the written consent is available for review by the Editor-in-Chief of this journal on request. In addition, all legal guardians were informed that they had a full right to discontinue the study without penalty. Confidentiality was kept, the questionnaire was anonymous, and data was presented without reflecting individual information.

## Source of funding

Not applicable.

## Author contribution

All authors developed the study design and data collection tool from previous literature. Ali has a research idea, arranged, and collected data, wrote a report and drafted a manuscript. Aden Conducted the Dermoscopic assessment, collected microscopic photos then reported to the senior dermatologist. Khalif analyzed the data, drafted a manuscript and developed the study time frame. All authors read, suggested corrections if needed and approved the manuscript.

## Conflicts of interest disclosure

All authors declare that they don’t have any conflicts of interest.

## Research registration unique identifying number (UIN)

Not applicable.

## Guarantor

Ahmed Isse Ali.

## Availability of data

All datasets generated and analyzed during the current study are included in this article.

## Provenance and peer review

Not commissioned, externally peer-reviewed.
